# Decoding the secretory blueprint of bone healing: from gradients to regeneration

**DOI:** 10.3389/fcell.2026.1873387

**Published:** 2026-06-30

**Authors:** Wenxiao Yang, Yuanfang Wang, Keda Liu, Yutong Zhao, Wei Wang

**Affiliations:** Liaoning Provincial Key Laboratory of Oral Diseases, School and Hospital of Stomatology, China Medical University, Shenyang, China

**Keywords:** bone regeneration, extracellular vesicles, immune microenvironment, secreted proteins, signaling crosstalk, spatiotemporal gradient

## Abstract

Bone tissue regeneration constitutes a complex biological process involving the coordinated regulation of multiple cell types and signaling pathways. As essential mediators of intercellular communication, secreted proteins play a pivotal role in directing mesenchymal stem cells (MSCs) recruitment, their differentiation into osteoblasts (OBs), the proliferation and activation of these OBs, as well as the synthesis and mineralization of the bone matrix. Furthermore, they modulate angiogenesis through dynamic alterations in concentration gradients, which are meticulously timed and spatially targeted, thereby facilitating their interconnected interactions. This precise regulation ultimately underpins the repair and regeneration of functional bone tissues. Given their critical regulatory function in regeneration, secreted proteins serve as key targets in bone tissue engineering and strategies aimed at repairing bone defects. In this review, we provide a systematic overview of recent advances in the research concerning secreted proteins in regulating osteogenesis, to provide novel insights and directions to further the development of bone regenerative medicine.

## 1 Introduction

Bone regeneration is a highly orchestrated process that depends on the coordinated action of multiple cell types, extracellular matrix (ECM) components, and signaling molecules. Over the past few decades, a large body of work has identified individual secreted proteins, including bone morphogenetic proteins (BMPs), fibroblast growth factors (FGFs), vascular endothelial growth factor (VEGF), and interleukins (ILs), as critical regulators of osteogenesis and bone repair. However, most existing reviews have focused on individual signaling pathways (e.g., BMP, Wnt) or single growth factor families, largely treating them in isolation. This work distinguishes itself by providing a unified, spatiotemporally organized framework that integrates multiple classes of secreted proteins—BMPs, FGFs, VEGF, interleukins, and emerging vesicular mediators—into the consecutive phases of inflammation, repair, and remodeling. Through this integrative perspective, the review decodes the secretory blueprint of bone healing and highlights how the timing, concentration gradients, and pathway crosstalk collectively govern regeneration, thereby offering a conceptual roadmap for designing next-generation multi-factor, phase-specific therapeutic strategies.

## 2 Osteogenesis

### 2.1 Proliferation and differentiation of osteoblast precursor cells

**FIGURE 1:**
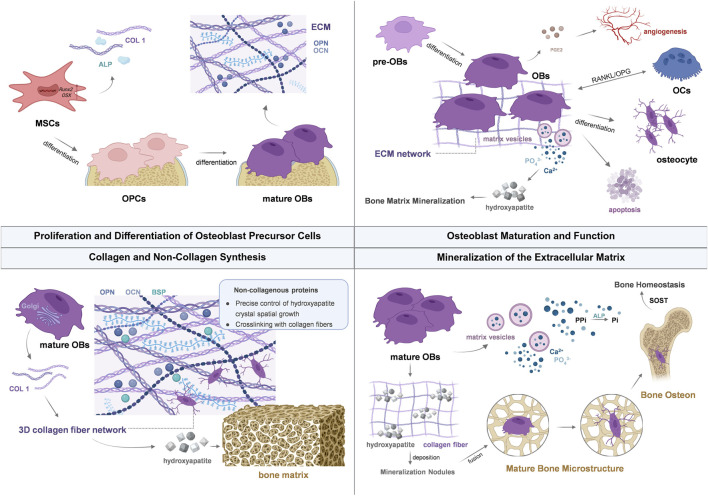
Schematic illustration of osteoblast-mediated bone formation and mineralization. This figure delineates the sequential process from mesenchymal stem cell (MSCs) differentiation to the formation of a mineralized bone matrix. Initiated by the co-expression of transcription factors RUNX2 and OSX, MSCs commit to the osteogenic lineage and differentiate into osteoblast pre-cursor cells (OPCs), characterized by the secretion of ALP and COL I. OPCs further mature into pre-osteoblasts and then into functional osteoblasts (OBs), marked by the upregulated expression of osteogenic genes (e.g., *SPP1, BGLAP*). Mature OBs extensively synthesize and secrete ECM components, predominantly COL I, which forms the structural scaffold. Concurrently, OBs release matrix vesicles rich in calcium, phosphorus, and ALP, which initiate mineralization by hydrolyzing PPi and facilitating the deposition of hydroxyapatite crystals along collagen fibers. Furthermore, OBs regulate bone remodeling via the RANKL/OPG signaling axis and promote vascularization through PGE2 release. Ultimately, the growing mineralized nodules mature into a structured bone matrix, wherein embedded OBs differentiate into osteocytes.

Bone tissue regeneration is a complex and highly organized biological process involving the interaction of multiple cell types and the precise regulation of essential growth factors (Figure 1) (Wang et al., 2022). Osteoblast precursor cells (OPCs) play a crucial intermediary role in the differentiation of mesenchymal stem cells (MSCs) into mature osteoblasts. Primarily situated within the bone marrow and the trabecular surfaces of bones, these cells possess a significant capacity for proliferation, rendering them indispensable for bone formation and repair. They are considered the fundamental cells responsible for osteogenesis (Rickard et al., 1996).

During the initiation of osteogenic differentiation, runt-related transcription factor 2 (*RUNX2*) is co-expressed with the osteogenesis-related transcription factor osterix, encoded by *SP7* (commonly referred to as OSX). This cooperation guides MSCs to differentiate into OPCs, a process marked by the production and release of alkaline phosphatase (ALP) and type I collagen (COL I). Subsequently, OPCs further mature into pre-osteoblasts—precursors to mature OBs—and exhibit a significant increase in osteogenic-specific gene expression (such as *SPP1* encoding osteopontin, and *BGLAP* encoding osteocalcin). This change triggers extensive synthesis and secretion of bone matrix components (Kim et al., 2020).

### 2.2 Osteoblast maturation and function

Osteoblasts (OBs) serve as the primary cells responsible for bone formation, undergoing a meticulously regulated, multi-stage maturation process. This process begins with pre-osteoblasts, progresses to fully mature and active osteoblasts, and ultimately results in either becoming osteocytes or undergoing programmed cell death. Mature OBs exhibit several defining features: a distinctive cellular morphology characterized by elongated membrane extensions that establish a communication network with adjacent osteocytes; elevated levels of ECM synthesis and secretion; peak activity of ALP and the hydrolysis of pyrophosphate to facilitate mineralization; and the targeted release of matrix vesicles that accumulate calcium and phosphorus ions. These vesicles play a crucial role in mobilizing calcium and phosphorus, directing the formation of hydroxyapatite crystals, and initiating the mineralization of the bone matrix (Kim et al., 2020).

Additionally, OBs modulate the bone microenvironment through two paracrine mechanisms: the receptor activator of nuclear factor-κB ligand (RANKL)/Osteoprotegerin (OPG) signaling axis, which dynamically regulates osteoclasts (OCs) activity and maintains the balance of bone remodeling; and the release of prostaglandin E2 (PGE2), which promotes vascularization to ensure the delivery of nutrients to bone tissues and facilitate repair processes (Chen et al., 2018).

### 2.3 Collagen and non-collagen synthesis

Mature OBs synthesize and secrete substantial quantities of ECM, with more than 90% of the bone’s organic matrix comprising COL I. Generated by OBs, COL I undergoes processing within the Golgi apparatus and is subsequently secreted externally, thereby establishing a three-dimensional network of collagen fibers that forms the structural framework of the bone matrix.

Non-collagen elements, such as osteopontin (OPN), osteocalcin (OCN), and bone sialoprotein (BSP), influence the functionality of bone tissue through two primary mechanisms: they meticulously regulate the spatial arrangement of hydroxyapatite crystals to improve mechanical strength, and they establish covalent cross-links with collagen fibers, thereby substantially enhancing the structural integrity of the bone. This covalent cross-linking with collagen fibers notably elevates the overall strength matrix of the bone (Kim et al., 2020).

### 2.4 Mineralization of the extracellular matrix

Bone matrix mineralization commences when OBs secrete matrix vesicles enriched with calcium, phosphorus ions, and enzymes such as ALP. These vesicles initiate mineralization through various mechanisms: ALP hydrolyzes inorganic pyrophosphate (PPi) to generate inorganic phosphate (Pi), thereby diminishing the inhibitory effect of PPi on mineralization; hydroxyapatite crystals are deposited along the axial direction of collagen fibers, forming initial mineralized nodules; these nodules grow through constrained expansion, eventually maturing into fully mineralized structures. They continue to grow and fuse via spatially limited expansion, remodeling into a highly organized mature bone microstructure. Ultimately, OBs become embedded within the mineralized matrix and differentiate into osteocytes. Osteocytes detect mechanical stimuli through the synaptic network and secrete factors such as sclerostin, which plays a crucial role in maintaining bone homeostasis and regulating mineral metabolism tissue (Murshed and McKee, 2010).

## 3 Secreted proteins

The process of bone healing—whether following fracture, surgical defect, or implantation—unfolds along a broadly conserved temporal axis: an initial inflammatory phase, a reparative phase characterized by soft and hard callus formation, and a prolonged remodeling phase. Secreted proteins are the principal mediators of this progression, but their roles are phase-specific and concentration-dependent. In this section, we profile the major families of secretory proteins, placing each in the functional context of the healing stage(s) it predominantly governs (Kim et al., 2020; Urist et al., 1983).

### 3.1 Temporal regulation mechanism of BMP family proteins

Bone morphogenetic protein (BMP), a constituent of the transforming growth factor-β (TGF-β) superfamily, functions as a vital inducer of osteogenic differentiation. It facilitates bone formation through multiple mechanisms: directly initiating the osteogenic program within MSCs, acting as a catalyst for bone matrix synthesis and mineralization, mediating the coupling between bone and blood vessels, and coordinating the spatial and temporal aspects to safeguard newly formed bone tissue. This coordination ensures the proper reconstruction of the new bone (Aryal et al., 2014; Xiao et al., 2007). Key BMP family members (BMP-2/4/5/6/7/9) regulate bone regeneration through dose-dependent, time-dependent pathways—starting with low concentrations that recruit MSCs and progenitor cells, followed by high-concentration signaling that drives osteogenic differentiation, then regulated mineralization, and finally constructing a functional microenvironment. This targeted approach underpins bone regenerative medicine.

During the early phase of injury, local cells—including inflammatory cells, periosteal cells, and endothelial cells—release low levels of BMPs, principally BMP-2 and BMP-7. These factors establish a concentration gradient that declines outward from the injury site. This gradient directs the chemotactic migration of peripheral MSCs toward the injury center and concurrently stimulates their local proliferation.

Recruited MSCs within the target area encounter lineage commitment locking under conditions of sustained local BMP production and elevated concentrations. BMPs effectively inhibit their differentiation into adipose or cartilage tissues while robustly activating core transcription factors such as RUNX2 and OSX via the classical Smad1/5/8 pathway, directing MSCs toward the osteogenic lineage. During the matrix pre-constructive stage, occurring concurrently, BMPs markedly enhance ALP activity and stimulate the synthesis of ECM components, thereby establishing the structural foundation necessary for subsequent mineralization.

As the process advances to bone matrix maturation and mineralization—primarily mediated by BMP-2/4—the role of BMPs transitions to regulating mineralization, as evidenced by the continued promotion of late-stage markers such as OCN and BSP, as well as supporting the differentiation of OBs into mature osteocytes, ultimately contributing to the formation of a functional bone homeostasis microenvironment (James et al., 2016; Bharadwaz and Jayasuriya, 2021; Wu et al., 2016).

Currently, the U.S. Food and Drug Administration (FDA) has authorized the use of BMP-2 and BMP-7 as adjunct therapies in spinal fusion procedures, the repair of bone defects, and the facilitation of bone union in cases of delayed fracture healing (Xiao et al., 2007; James et al., 2016; Salazar et al., 2016). The FDA has approved a BMP-2 concentration of 1.5 mg/mL for human use, recognized as the optimal quantity for stimulating new bone growth (James et al., 2016). BMP-7 promotes hydroxyapatite crystal formation by lowering pro-inflammatory cytokines like TNF-α and IL-1β during bone healing. It also aids OBs activity to mineralize deposits *in vitro* and enhances bone regeneration (Wiesli et al., 2025). These characteristics highlight its clinical importance in spinal fusion procedures and treatment of fracture nonunion.

**FIGURE 2:**
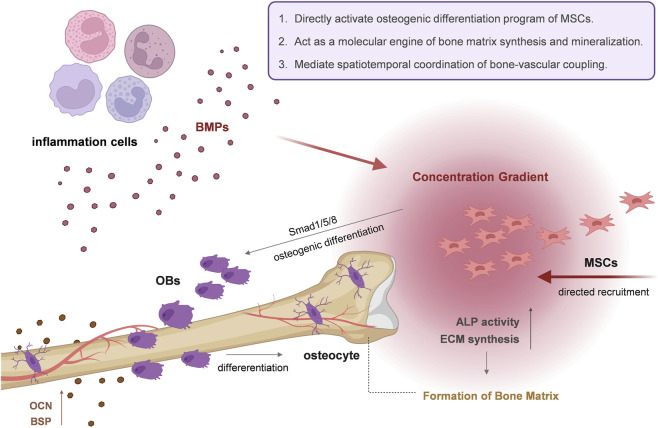
Temporal and dose-dependent regulation of osteogenesis by BMP signaling. This illustration outlines the multi-stage role of Bone Morphogenetic Proteins (BMPs) in bone regeneration. Following injury, a low-concentration BMP gradient recruits mesenchymal stem cells (MSCs) to the site. Sustained, high local BMP concentrations then direct osteogenic lineage commitment by activating key transcription factors via the Smad1/5/8 pathway, while inhibiting alternative fates. During the matrix pre-construction phase, BMPs enhance ALP activity and stimulate the synthesis of ECM components. Subsequently, BMPs promote matrix maturation and mineralization by upregulating late markers (e.g., OCN, BSP) and supporting osteocyte differentiation.

The dose- and time-dependent regulatory logic described above is schematically summarized in Figure 2, which illustrates the progressive transition from low-concentration BMP-mediated MSC recruitment to high-concentration osteogenic commitment, matrix construction, and eventual mineralization.

### 3.2 Time-delayed modulation pattern of FGF signaling

The fibroblast growth factor (FGF) family functions as a “multi-stage molecular coordinator’ throughout the process of bone regeneration by binding to and activating its receptor isoforms (FGFR1-4) in a spatiotemporal manner. It is indispensable at each stage: during the initial phase, it promotes the mitosis of MSCs and vascularization (for instance, FGF-2 exhibits potent chemotactic and pro-proliferative effects); in the intermediate phase, it facilitates granulation tissue formation and the development of bone matrix (for example, FGF-18 influences osteogenesis within cartilage); and in the final phase, it guarantees the quality of bone regeneration and sustains bone homeostasis (such as FGF-23, which modulates phosphorus metabolism homeostasis) (Charoenlarp et al., 2017). This dual regulation, which depends on dosage and timing, enables FGFs to precisely coordinate the entire bone regeneration process.

During the initial inflammatory phase, FGF-2 released after injury serves as a potent chemokine that attracts inflammatory cells such as macrophages and neutrophils, as well as MSCs, to the injury site. It promotes the initial proliferation of local MSCs and periosteum-derived stem/progenitor cells, thereby enhancing their potential for differentiation into osteogenic and chondrogenic lineages. Furthermore, FGF-2 stimulates the production of matrix metalloproteinases (MMPs), which are critical for bone regeneration. It induces the expression of MMP-9, thereby facilitating ECs proliferation, migration, and neovascularization to support blood supply during the repair process. Additionally, FGF-2 facilitates the transition from inflammation to repair by promoting macrophage M2 phenotypic polarization (Charoenlarp et al., 2017; Zhou et al., 2023; Yang et al., 2018; Zhang et al., 2020). Animal studies have demonstrated that the combination of FGF-2 with MSCs transplantation enhances osteogenesis in femoral fracture regions and expedites bone tissue regeneration (Zhou et al., 2023; Takeda et al., 2024).

During the intermediate repair phase, members of the FGF family—principally FGF-2, FGF-9, and FGF-18—collaborate to promote granulation tissue formation and scab development, while meticulously regulating cellular proliferation, differentiation, and matrix synthesis. FGF-2, a potent mitogen, significantly enhances the proliferation of OPCs, periosteal cells, and chondrocytes. It also functions synergistically with FGF-9 to elevate levels of VEGF and angiogenin 2 in ECs. FGF-18 plays a critical role in the osteogenesis of cartilage by activating the FGFR3 receptor and downstream MAPK and PI3K-Akt signaling pathways; it further supports chondrocyte growth and cartilage matrix formation in concert with FGF-9. Additionally, FGF-18 inhibits autophagy in hypertrophic chondrocytes through FGFR4, stabilizes collagen type II (COL II) secretion, and promotes proper cartilage template development, thereby intricately regulating the process of endochondral ossification (Charoenlarp et al., 2017; Wang et al., 2017; Murugaiyan et al., 2023; Cinque et al., 2015).

During the late stage of remodeling, FGF-23 functions as a vital hormone for phosphorus regulation by providing negative feedback to constrain bone mineralization. It achieves this by diminishing renal phosphorus reabsorption and reducing active vitamin D levels, which collectively assist in decreasing blood phosphorus levels and maintaining mineralization equilibrium. Upon the completion of cartilage osteogenesis, pro-chondrogenic signals such as FGF-9 and FGF-18 exhibit a marked decline. Conversely, osteogenic signals—including the BMP/Smad pathway and Wnt/β-catenin pathway—and bone turnover signals such as the RANKL/OPG pathway become more dominant in the process of bone remodeling (Murugaiyan et al., 2023; Moe and Moe, 2017).

**FIGURE 3:**
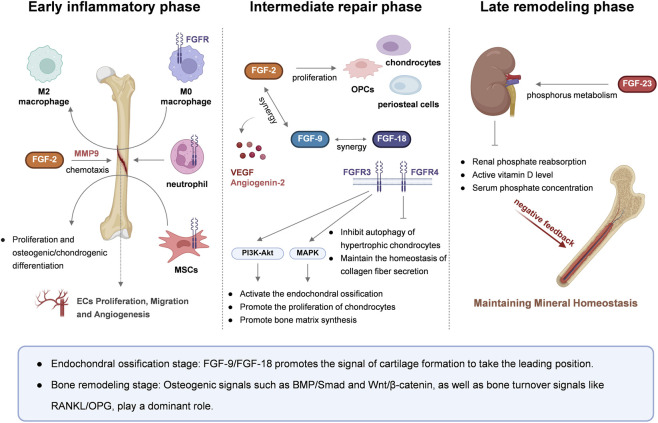
Time-delayed modulation of bone regeneration by FGF signaling. This figure illustrates the stage-specific coordination of bone repair by distinct FGF family members. During the initial inflammatory phase, FGF-2 acts as a potent chemoattractant for inflammatory cells and mesenchymal stem cells (MSCs), promotes their proliferation, and induces macrophage polarization towards the M2 phenotype to transition from inflammation to repair. It also stimulates MMPs expression and angiogenesis. In the intermediate repair phase, FGF-2, FGF-9, and FGF-18 collaboratively drive cellular proliferation and matrix synthesis. FGF-2 and FGF-9 synergistically enhance angiogenic fac-tors, while FGF-18 (via FGFR3) and FGF-9 promote chondrocyte growth and cartilage matrix formation for endochondral ossification. During the late remodeling phase, FGF-23 acts as a systemic hormone to inhibit mineralization by regulating phosphate homeostasis, while pro-chondrogenic signals decline, allowing osteogenic pathways (e.g., BMP, Wnt) to dominate bone remodeling and maintain homeostasis.

Figure 3 integrates these phase-specific functions into a unified timeline, highlighting the sequential involvement of FGF-2 (early inflammation and angiogenesis), FGF-9/18 (intermediate chondrogenesis and matrix synthesis), and FGF-23 (late systemic mineral homeostasis) as bone healing progresses.

### 3.3 VEGF: a key regulator of vascularized bone regeneration

Vascular endothelial growth factor (VEGF) is a highly specific mitogen for vascular ECs and plays a pivotal role in coordinating angiogenesis and osteogenesis during bone regeneration. As a primary driver, VEGF facilitates the restoration of the regenerative microenvironment through a triple cascade mechanism: promoting angiogenesis, activating osteogenesis, and recruiting stem cells (Hu and Olsen, 2016).

Angiogenesis represents the initial stage in the triple cascade process responsible for establishing a vascular network essential for bone development, thereby providing fundamental structural support and a suitable microenvironment. During this phase, VEGF-A, the predominant isoform, promotes the growth of new blood vessels by binding to VEGFR2 receptors on ECs, which induces the release of MMPs. This activity guides ECs migration toward hypoxic or damaged regions through the degradation of basement membranes and ECM components. Simultaneously, it facilitates the delivery of oxygen and nutrients to OBs and encourages ECs to secrete paracrine factors such as PDGF-BB, which recruit perivascular cells. These combined processes collaboratively contribute to structural repair and support the maturation and mineralization of bone tissue (Hu and Olsen, 2016; Pérez-Gutiérrez and Ferrara, 2023; Kaigler et al., 2006).

At the stage of osteogenic activation, VEGF converts angiogenic signals into bone formation signals, establishing “vascular-osteogenic coupling.” VEGF activates the Smad1/5/8 pathway in MSCs and OPCs by inducing osteogenic factors, primarily BMP-2, and directly promotes osteogenic differentiation. It can also directly facilitate the differentiation of MSCs into OBs via VEGFR1 and VEGFR2 signaling pathways, while additionally enhancing the proliferation, survival, and mineralization of mature OBs. Furthermore, the newly formed bone matrix releases BMPs and TGF-β, which further stimulate VEGF expression, thereby creating a positive feedback loop (Hu and Olsen, 2016; Kusumbe et al., 2014; Tang et al., 2021). Research indicates that bone marrow mesenchymal stem cells (BMSCs) composite scaffolds, modified through the co-expression of BMP-2 and VEGF, substantially augment osteogenesis in critical cranial defects, thereby confirming the synergistic regulatory effects of VEGF and bone metabolism factors (Guo et al., 2023).

VEGF’s ability to recruit stem cells provides a cellular source for ongoing bone regeneration: it functions as a chemokine that attracts VEGFR1 on MSCs, thereby directly facilitating their migration toward hypoxic or damaged regions. Moreover, VEGF promotes the secretion of stromal cell-derived factor 1 (SDF-1, also known as CXCL12) by ECs and bone stromal cells, guiding MSCs with elevated levels of CXCR4 to migrate along the SDF-1 gradient toward the site of bone formation (Peng et al., 2020; Grunewald et al., 2006). These three interconnected processes establish positive feedback loops, collectively creating a self-sustaining microenvironment conducive to bone repair.

**FIGURE 4:**
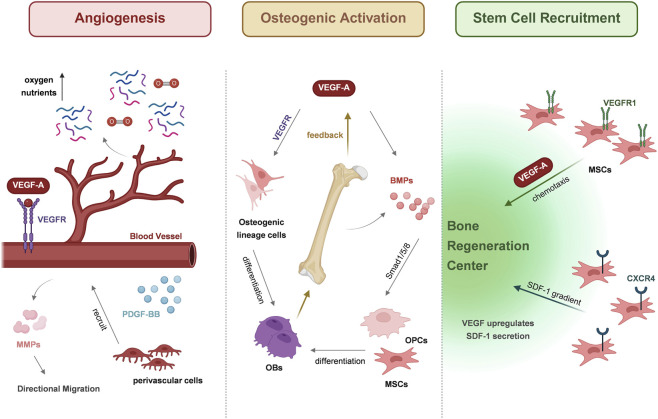
VEGF-mediated vascularized bone regeneration through a triple cascade mechanism. This figure illustrates the pivotal role of Vascular Endothelial Growth Factor (VEGF) in coordinating angiogenesis and osteogenesis. VEGF orchestrates bone repair through three interconnected processes: 1) Angiogenesis: VEGF-A binding to VEGFR2 on endothelial cells (ECs) induces matrix metalloproteinase (MMP) release, guiding ECs migration and tubulogenesis to form new blood vessels, which deliver oxygen and nutrients. These ECs also secrete factors like PDGF-BB to recruit peri-vascular cells for vessel maturation. 2) Osteogenic Activation: VEGF establishes “vascular-osteogenic coupling” by inducing BMP-2 to activate the Smad1/5/8 pathway in mesenchymal stem cells (MSCs) and osteoblast precursor cells (OPCs), directly promoting osteogenic differentiation and mature osteoblast function. A positive feedback loop is formed as the new bone matrix releases factors that further stimulate VEGF ex-pression. 3) Stem Cell Recruitment: VEGF acts as a chemoattractant by binding to VEGFR1 on MSCs and stimulates ECs to secrete SDF-1, which guides CXCR4-expressing MSCs to the injury site. These three cascades create a self-sustaining microenvironment that drives vascularized bone regeneration.

The triple cascade mechanism—angiogenesis, osteogenic activation, and stem cell recruitment—through which VEGF coordinates vascularized bone repair is summarized in Figure 4, emphasizing the positive feedback loops that sustain the regenerative microenvironment.

### 3.4 ILs: mediators that regulate inflammation on both sides during bone regeneration

Interleukins (ILs) are vital cytokines that regulate the immune-inflammatory response and demonstrate a dynamic “pro-inflammatory-repair biphasic transition” during bone regeneration. Initially, pro-inflammatory isoforms (IL-1β/IL-6/IL-17) predominate, facilitating the clearance of necrotic tissue and defending against pathogens. In subsequent phases, reparative isoforms (IL-4/IL-10/IL-13) promote osteogenic differentiation and matrix formation by inhibiting the NF-κB pathway and encouraging M2 macrophage polarization (Ni et al., 2022; Lee, 2013; Zhang et al., 2014; Takeuchi et al., 2021). The meticulous regulation of the spatiotemporal expression of ILs is essential for balancing inflammation and regeneration, thereby facilitating effective bone reconstruction.

During the initial pro-inflammatory phase, activated M1 macrophages, neutrophils, and osteocytes secrete substantial quantities of IL-1, predominantly IL-1β. IL-1 amplifies the inflammatory response by robustly activating the NF-κB pathway and inducing other pro-inflammatory mediators such as IL-6, TNF-α, chemokines, and MMPs. Concurrently, IL-1 directly inhibits OBs proliferation, differentiation, and mineralization, while markedly promoting osteoclastogenesis and bone resorption through the upregulation of RANKL (Polzer et al., 2010; Tseng et al., 2022). As the process transitions to healing, IL-4 and IL-13, produced by Th2 cells, M2 macrophages, and basophils, serve as key regulatory cytokines. They induce macrophages to transition from the pro-inflammatory M1 phenotype to the anti-inflammatory, repair-oriented M2 phenotype and diminish the production of pro-inflammatory factors by M1. Furthermore, IL-4 and IL-13 facilitate OBs proliferation and differentiation through the STAT6 pathway and suppress osteoclastogenesis by reducing RANKL expression (Iwaszko et al., 2021; Allen, 2023). The equilibrium between pro- and anti-inflammatory signals, alongside the suppression of inflammation and local microenvironmental cues, is crucial for a seamless transition.

This “pro-inflammatory-repair biphasic transition” mediated by the IL family ensures that the initial inflammatory response effectively eliminates damage and pathogens, then shifts to the bone repair phase in a timely manner. This process helps prevent pathological bone destruction or fibrosis caused by chronic inflammation. Studies into molecular mechanisms confirmed that MSC spheroid cultures modified with the *IL-4* gene showed significantly improved chondroprotective and anti-inflammatory effects in an *in vitro* osteoarthritis (OA) model, and successfully promoted cartilage regeneration in OA rats (Song et al., 2020). Research showed that macrophages treated with immunomodulatory nanoparticles significantly improved MSCs’ osteogenic differentiation by encouraging M2 polarization via the IL-10/STAT3 signaling pathway (Mahon et al., 2020).

**FIGURE 5:**
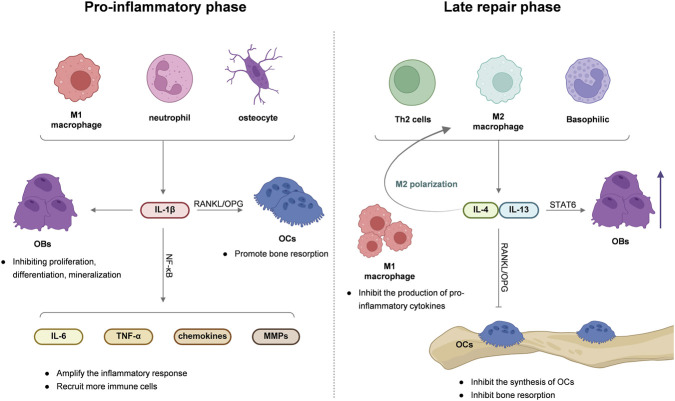
Biphasic regulation of bone regeneration by ILs. This illustration illustrates the dynamic and biphasic role of interleukins (ILs) in coordinating the transition from inflammation to repair during bone healing. During the initial pro-inflammatory phase, IL-1β secreted by M1 macrophages and other immune cells, activate the NF-κB pathway. This promotes inflammation, recruits immune cells, and initiates bone resorption by upregulating RANKL while simultaneously inhibiting osteoblast function. As healing progresses, a transition to the reparative phase occurs, driven by IL-4 and IL-13. These cytokines induce a shift from pro-inflammatory M1 to reparative M2 macrophage polarization, inhibit NF-κB signaling, and directly promote osteoblast proliferation and differentiation via pathways like STAT6. This carefully orchestrated biphasic transition ensures that inflammation effectively clears debris and pathogens before resolving to allow for matrix formation and bone regeneration, preventing chronic inflammation and facilitating successful tissue reconstruction.

The biphasic transition from IL-1β-driven early inflammation to IL-4/IL-13-mediated reparative macrophage polarization and osteoblast activation is illustrated in Figure 5, underscoring the importance of timely resolution of inflammation for successful bone regeneration.

### 3.5 Extracellular vesicles and niche-derived signals: key emerging mediators of the osteoregenerative secretome

Beyond classical soluble proteins, the osteoregenerative secretome comprises a broader repertoire of intercellular mediators—particularly extracellular vesicles (EVs) and signals derived from specialized niche-resident cells—whose functional importance in bone repair has only recently been elucidated. Among these, EVs—including exosomes and microvesicles—serve as sophisticated, cargo-loaded signaling platforms secreted by mesenchymal stromal cells, OBs, and other constituents of the bone microenvironment (Huang et al., 2020). Bone-targeting engineered exosomes derived from iPSC-derived MSCs and loaded with siRNA targeting Schnurri-3 (*SHN3*) siRNA have been shown to specifically silence the osteoblastic *SHN3* gene, thereby enhancing osteogenic differentiation, promoting type H vessel formation through SLIT3 upregulation, and suppressing osteoclastogenesis via reduced RANKL expression, exemplifying a multi-pronged, EV-mediated approach to osteoporotic bone repair (Cui et al., 2021).

Concurrently, the cellular niche within the healing bone—comprising osteomacs, CXCL12-abundant reticular (CAR) cells, and vascular endothelial cells—functions as a spatially constrained signaling hub. Within this hub, classically activated M1-like osteomacs have been shown to secrete Oncostatin M (OSM) through a COX2-PGE2 regulatory loop; OSM then binds to the OSMR-gp130 receptor complex on MSCs, activating a STAT3-C/EBPδ-RUNX2 signaling cascade that potently drives osteogenic differentiation and matrix mineralization, thereby directly coupling innate immune activation to bone formation (Guihard et al., 2012). In addition to the osteomac-derived OSM–STAT3–C/EBPδ–RUNX2 axis, the BMP-2–driven differentiation of CXCL12-abundant reticular (CAR) cells from local mesenchymal precursors provides a complementary cellular route through which the healing niche couples stromal organization with osteogenesis (Tosa et al., 2025). Collectively, these niche-derived signals confer topographic precision to regenerative cues—transforming broadly distributed protein gradients into spatially restricted, context-dependent instructions that ensure targeted activation of repair programs.

### 3.6 Protein trafficking in the three-dimensional bone microenvironment

In the mineralized three-dimensional bone microenvironment, secreted protein transport extends far beyond passive diffusion and is instead tightly regulated by integrated physical, cellular, and biochemical mechanisms. First, the highly organized, interwoven architecture of collagen fibrils and hydroxyapatite nanocrystals forms a dense, nanoscale porous matrix that functions as a molecular sieve—imposing size- and charge-selective constraints on diffusion: small proteins diffuse slowly, whereas larger protein aggregates or complexes are effectively retained. Beyond this physical barrier, numerous secreted proteins, including OPN and OCN,bind directly to hydroxyapatite surfaces via clustered acidic amino acid motifs, or to COL I fibrils; such binding is not merely incidental but constitutes a functionally essential step in matrix maturation and mineral nucleation (Komori, 2020; Bai et al., 2022). Concurrently, physiological mechanical loading induces interstitial fluid flow through the lacunar-canalicular network, generating convective transport that facilitates rapid, directional movement of select proteins across micrometer-scale canaliculi (García-Aznar et al., 2021). Collectively, these transport modalities establish the structural confinement and kinetic framework necessary for morphogen gradient formation; however, their inherent lack of molecular specificity means they cannot, by themselves, explain the spatially precise, molecule-specific distribution patterns observed for distinct signaling factors.

Glycosaminoglycans (GAGs) —a class of linear, negatively charged polysaccharides—are essential structural and functional constituents of the bone ECM. They occur either as free polysaccharide chains or as covalently attached side chains on core proteins to form proteoglycans, including heparan sulfate proteoglycans (HSPGs) (Khan et al., 2024). Heparan sulfate (HS), a highly sulfated GAG, functions as a master regulatory scaffold in bone repair: its variably sulfated domains bind with high affinity and selectivity to key secreted signaling proteins, such as BMP-2, VEGF, and FGFs, thereby controlling their bioavailability, spatial distribution, and stability. Specifically, HS binding sequesters these morphogens from bulk diffusion, enriches them at cell surfaces or within discrete ECM microdomains, and shields them from proteolytic cleavage. Critically, the ‘sulfation code’ —defined by the precise location, type (N-, 2-O-, 6-O-sulfation), and density of sulfate groups along the HS chain—determines ligand specificity: distinct sulfation motifs selectively recruit particular morphogens, thereby establishing spatially restricted enrichment zones and modulating local retention strength (Li et al., 2025). Moreover, HS does not merely act as a passive reservoir; it serves as a co-receptor that facilitates the formation of high-affinity ternary signaling complexes (e.g., FGF-FGFR-HS), thereby potentiating both the amplitude and duration of downstream signal transduction (Naimy et al., 2011). In parallel, hyaluronic acid (HA), another major GAG in bone, exhibits a molecular weight—dependent functional duality: high-molecular-weight HA (HMW-HA) contributes to the structural integrity and quiescence of stem cell niches, whereas injury- or inflammation-induced low-molecular-weight HA fragments (LMW-HA) actively stimulate angiogenesis and osteogenic differentiation (Moreno et al., 2023; Lee-Sayer et al., 2015). Collectively, through these integrated biophysical and biochemical mechanisms, GAGs convert the inherently nonspecific diffusion environment of the mineralized ECM into a dynamically regulated, spatially instructive signaling landscape—precisely sculpting concentration gradients and signal intensities of secreted morphogens to guide tissue regeneration.

**FIGURE 6:**
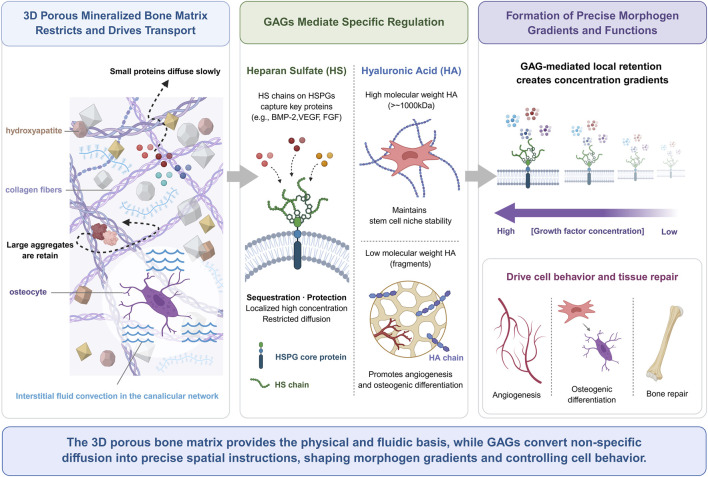
Secretory protein trafficking and its regulation by glycosaminoglycans in the 3D bone microenvironment. In the mineralized bone matrix, passive transport is governed by molecular sieving of the collagen–hydroxyapatite network, direct protein binding to hydroxyapatite, and interstitial fluid flow-driven convection. HS chains selectively capture morphogens through sulfation-dependent affinity, enabling localized enrichment and slow release. HA exerts molecular-weight-dependent dual effects: HMW-HA maintains niche stability, while LMW-HA fragments promote angiogenesis and osteogenesis. Collectively, these GAG-mediated mechanisms transform passive diffusion into spatially defined morphogen gradients that direct cell differentiation.

Within this GAG-regulated transport system, morphogens—including BMPs and Wnts—are spatially organized into steep, boundary-defined concentration gradients upon reaching MSCs, thereby directly instructing their osteogenic lineage commitment and subsequent ECM mineralization. Disruption of this regulatory system precipitates profound pathological outcomes: in aging, osteoporosis, and chronic inflammatory conditions, altered HS sulfation patterns or imbalanced HA fragment generation impair gradient integrity, diminish signaling fidelity, and skew MSC differentiation toward non-osteogenic fates—ultimately compromising bone repair capacity (Li et al., 2025). The mechanistic understanding of GAG-mediated morphogen trafficking has thus catalyzed the emergence of ‘traffic engineering’ as a paradigm for bone regeneration. In this approach, GAG-functionalized biomaterials are rationally engineered to recapitulate key biophysical features of native ECM—specifically, the sulfation code of HS and the molecular weight-dependent bioactivity of HA—to restore physiologically appropriate spatiotemporal distributions of endogenous or delivered growth factors (Mansour et al., 2017). By reconstituting therapeutic morphogen gradients with precise spatial control and kinetic stability, these strategies hold significant promise for accelerating the regeneration of critical-size bone defects. Figure 6 provides a schematic overview of the transport and regulatory mechanisms discussed in this section.

## 4 Essential signaling pathways in osteogenesis

### 4.1 Wnt signaling pathway: biphasic regulatory effects

As introduced in Section 2, the Wnt family exemplifies the biphasic regulatory logic that governs osteogenesis. Here, we elaborate on the molecular architecture of both canonical and non-canonical Wnt signaling and their integration with mechanical cues.

The Wnt family of proteins functions as key regulators of osteogenic differentiation, precisely orchestrating bone regeneration via both the classical (β-catenin-dependent) and non-classical (β-catenin-independent) pathways (Tan et al., 2019). During osteogenic differentiation, Wnt signaling demonstrates a distinctive biphasic expression pattern: it remains at low levels initially to prevent premature osteogenesis and excessive inflammation, then significantly increases at a later stage to activate the β-catenin/TCF4 complex. This activation facilitates the differentiation of MSCs into bone-forming cells and promotes the synthesis of the bone matrix. Such biphasic regulation is essential for the spatial and temporal control of bone regeneration; early inhibition prevents untimely mineralization prior to the establishment of cellular scaffolds and sufficient cell numbers, while subsequent activation provides a strong impetus for efficient bone formation at the appropriate location and time.

During the initial phase of osseous healing, referred to as the inflammatory stage, macrophages and various other cells perform the clearance of necrotic tissue and secrete a substantial amount of inflammatory mediators, including TNF-α, IL-1β, and IL-6. Concurrently, MSCs are recruited to the injury site and commence proliferation, resulting in the formation of either a cartilaginous bone crust via endochondral ossification or a fibrous bone crust through intramembranous ossification. During this phase, inflammatory cytokines—particularly TNF-α—play a pivotal role in upregulating key Wnt pathway antagonists such as Dickkopf-related protein 1 (Dkk1, encoded by *DKK1*) and sclerostin (Sost, encoded by *SOST*). This transient inhibition is essential: without it, premature or excessive Wnt signaling could drive MSCs that are destined to form cartilage templates to differentiate prematurely into osteoblasts, risking repair failure or abnormal healing. Moreover, controlled suppression of Wnt signaling moderates the intensity and duration of the inflammatory response, thereby preventing excessive or prolonged inflammation and fostering optimal tissue regeneration (Huybrechts et al., 2020; Gao et al., 2023; Baron and Kneissel, 2013).

As inflammation diminishes and a cartilage or fibrous bone scab develops, the bone repair process reaches a pivotal stage characterized by the formation of a hard bony cap and the mineralization of the bone matrix. During this phase, inhibitors such as Dkk1 and Sost levels decrease significantly, while OPCs and OBs begin secretion of Wnt ligands (e.g., Wnt3a, Wnt10b), which activate the classical Wnt signaling pathway. These ligands bind to Frizzled receptors and LRP5/6 co-receptors on the cell surface, inhibiting the degradation complex responsible for phosphorylating and degrading intracytoplasmic β-catenin. Consequently, β-catenin accumulates in the cytoplasm and translocates into the nucleus. Inside the nucleus, β-catenin associates with TCF/LEF transcription factors, thereby promoting the expression of critical osteogenic genes such as *RUNX2* and *OSX* and initiating osteogenic differentiation (Huybrechts et al., 2020; Gao et al., 2023; Baron and Kneissel, 2013). This highly regulated spatiotemporal switch is crucial for proper and effective bone regeneration.

Wnt signaling exhibits a high responsiveness to mechanical stimuli, being under the meticulous regulation of multidimensional ligand expression, receptor activity, intracellular signaling pathways, and the surrounding microenvironment. Mechanical stimulation, including fluid shear stress, significantly enhances the secretion of Wnt1/3a and other ligands, activates downstream signaling cascades, and facilitates the translocation of β-catenin into the nucleus. Co-receptors LRP5/6 directly perceive mechanical stimuli, augment their binding affinity to Wnt ligands through conformational alterations, and inhibit the degradation of β-catenin. Additionally, mechanical loading induces osteocytes to release PGE_2_, which subsequently activates the classical Wnt signaling pathway via autocrine/paracrine mechanisms, thereby fostering bone formation (Kang and Robling, 2015; Kitase et al., 2010).

The functional significance of this biphasic Wnt regulation is further amplified by its extensive crosstalk with the BMP/Smad pathway (see 3.2) and the RANKL/OPG system (see 3.4), as discussed below.

### 4.2 BMP/smad signaling pathway: networked regulatory architecture

Building on the dose- and time-dependent roles of BMP family proteins described in Section 2.1, this section details the intracellular Smad-dependent transduction cascade and its crosstalk with parallel pathways. The BMP/Smad signaling pathway, a vital transduction pathway for BMPs, carefully governs the entire process of bone regeneration. It orchestrates the systematic transformation of MSCs into functional bone tissue via a mechanism that is precisely regulated in both spatial and temporal dimensions.

Following a bone injury, local BMPs establish a concentration gradient that diminishes from the site of injury outward. This gradient activates type I/II serine-threonine kinase receptors on the surface of periosteal stem cells and BMSCs. Upon activation, BMP receptors phosphorylate the receptor-regulated Smads (R-Smads: Smad1/5/8). Phosphorylated R-Smads then assemble into a heterotrimeric complex with the common mediator Smad4. This complex translocates into the nucleus, where it binds to GC-rich regions within the RUNX2/OSX promoter to promote osteogenic gene expression and initiate osteogenic differentiation. Continued BMP signaling stimulates these differentiating osteoblasts to produce additional BMPs, establishing an autocrine/paracrine positive feedback loop that amplifies and sustains osteogenic signaling (Wu et al., 2016; Wang et al., 2010).

During the process of bone matrix mineralization and maturation, inhibitory Smads (I-Smads: Smad6/Smad7) regulate signaling precision through negative feedback mechanisms. Smad7 competes with BMP type I receptors (such as ALK2/3/6) to inhibit the recruitment and phosphorylation of R-Smads, while Smad6 prevents the formation of the R-Smad/Smad4 transcription complex. Both Smad6 and Smad7 function as scaffolds, attracting E3 ubiquitin ligases (including Smurf1/2), which target R-Smads for ubiquitination and subsequent degradation. Additionally, Smad6 has been demonstrated to obstruct the BMP signaling route, whereas Smad7 can impede both the BMP and TGF-β signaling pathways. These combined effects are essential in preventing excessive mineralization and heterotopic ossification, thereby supporting homeostasis during tissue regeneration. Conversely, abnormal expression levels of I-Smads have been associated with conditions such as osteosclerosis and heterotopic ossification, underscoring their potential as therapeutic targets in bone regeneration (Zou et al., 2021; de Ceuninck van Capelle et al., 2020; Fang et al., 2016; Miyazawa and Miyazono, 2017).

During osteogenic differentiation and bone regeneration, the BMP/Smad and Wnt/β-catenin pathways do not operate independently; instead, they constitute a complex regulatory network characterized by intricate interactions. This network functions throughout all stages of bone regeneration—namely, initiation, progression, and maturation—serving to regulate and precisely coordinate the entire process. At the transcriptional level, these pathways exhibit a pronounced synergistic effect: both directly or indirectly elevating the expression of the key transcription factor RUNX2. Significantly, activated Smad1/5/8 proteins within the nucleus interact with β-catenin to collaboratively activate the RUNX2 promoter. The combined effects enhance downstream osteogenic gene transcription activity far more than the activation of an individual pathway, hence considerably boosting signals for osteogenic differentiation. Furthermore, the pathways mutually regulate each other by augmenting ligand and receptor expression as well as signaling output. Through temporal coordination, they assume dominant or supportive roles at various stages of bone regeneration, thereby ensuring a systematic process (Kim et al., 2021; Kaur et al., 2021; Pang et al., 2022; Rawadi et al., 2003; Mbalaviele et al., 2005).

Furthermore, TGF-β ligands (TGF-β1/2/3) influence bone regeneration in a biphasic manner by activating the Smad2/3 pathway: initially, increased levels of Cyclin D1 and MMP-2/9 promote OPCs proliferation and migration; subsequently, the competitive diminution of Smad4 inhibits BMP/Smad1/5 signaling, and the upregulation of the Sox9 transcription factor impedes osteogenic terminal differentiation (Wu et al., 2016; Wu et al., 2024).

The pathological counterpart of spatially dysregulated BMP signaling is heterotopic ossification (HO). In HO, abnormal signaling pathways—such as mutations in the *ACVR1/ALK2* genes that alter BMP type I receptor responsiveness—and changes in the microenvironment—like the release of inflammatory cytokines including TNF-α, IL-1, and IL-6—lead to elevated BMP levels. These increased BMPs strongly promote MSCs or multipotent progenitor cells (MPPs) in skeletal muscle mesenchyme to become OBs, causing ectopic bone formation (James et al., 2016; Wu et al., 2024; Wosczyna et al., 2012). In a tendon-specific mice model, the activation of ALK2 receptor expression induced spontaneous ectopic ossification within the Achilles tendon. This demonstrates that excessive activation of the BMP pathway in the microenvironment of the tendon directly results in ectopic endochondral ossification (Salazar et al., 2016; Yamaguchi et al., 2023). HO thus serves as a paradigmatic pathophysiological model illustrating the critical necessity for precise spatiotemporal control—and quantitative calibration—of BMP signaling. These fundamental principles directly inform the rational design of next-generation therapeutic BMP delivery systems for targeted bone regeneration.

As noted in 3.1, BMP/Smad signaling converges with Wnt/β-catenin at multiple levels—most notably through Smad1/5/8–β-catenin co-activation of the RUNX2 promoter—while also intersecting with MAPK-mediated RUNX2 phosphorylation and feeding into the RANKL/OPG axis during remodeling initiation.

### 4.3 MAPK pathway: mechanistic signal transduction processes

Cells within bone tissue consistently detect mechanical stimuli produced by weight-bearing, movement, implants, or external loading apparatus. The MAPK signaling pathway functions as a crucial center for mechanical signal transduction, reacting to mechanical signals in the microenvironment via a “mechanical sensing-signal cascade transmission” process, thus facilitating the transformation of mechanical stimuli into osteogenic effects.

In the process of bone regeneration, cells detect mechanical signals within the microenvironment via several essential receptors. Integrins (ITG) function as a critical link for the transmission of extracellular mechanical stimuli to the intracellular environment, facilitating the connection between the ECM and the cytoskeleton. Mechanical stimulation induces conformational changes in integrins, leading to the recruitment of focal adhesion kinase (FAK), Src, and additional proteins to establish signaling complexes. Ion channels, including Piezo1 and Piezo2, are directly activated by membrane tension, resulting in the influx of calcium ions. Furthermore, cytoskeletal networks, including actin microfilaments and microtubules, are capable of directly transmitting and sensing tension (Jang et al., 2022; Qin et al., 2021; Zhang et al., 2023; Hu et al., 2024).

Upon mechanical stimulation, these sensors trigger a cascade of early signaling events: integrin clustering induces the autophosphorylation and reciprocal activation of FAK and Src; the GRB2-SOS complex activates the small GTPase Ras; and stretch-activated ion channels mediate Ca^2+^ influx, which in turn activates Ca^2+^/calmodulin-dependent kinases such as CaMK (Miyazawa and Miyazono, 2017; Kim et al., 2021; Kaur et al., 2021; Pang et al., 2022). The initial signals converge and robustly activate the core cascade reactions of the downstream MAPK signaling pathway: Ras → Raf → MEK → ERK. This process culminates in the activation and nuclear translocation of ERK1/2, which subsequently activates downstream transcription factors such as RUNX2, OSX, and c-Fos/c-Jun, thereby promoting the expression of genes associated with osteoblast proliferation, differentiation, and bone matrix synthesis (Dou et al., 2019). Moreover, mechanical stimuli notably activate the p38 pathway (TAK1 → MKK3/6), which is essential for facilitating bone tissue formation, mineralization, and adaptive remodeling (Thouverey and Caverzasio, 2012).

The MAPK/ERK cascade does not operate independently: it potentiates Wnt signaling through LRP6 phosphorylation and directly activates RUNX2 in parallel with the BMP/Smad pathway, thereby serving as a convergent node for mechanical and growth factor inputs.

### 4.4 RANKL/OPG system: pathological imbalance regulation mechanism

The RANKL/OPG system is crucial for regulating OCs formation, activation, and survival during bone remodeling. OBs and osteocytes predominantly secrete the RANKL, which interacts with the receptor activator of nuclear factor-κB (RANK) on OCs precursors, thereby initiating the NF-κB and MAPK signaling pathways. This activation results in the induction of the essential transcription factor NFATc1, thereby promoting OCs differentiation and maturation (Honma et al., 2021). OPG, a soluble decoy receptor produced by OBs, binds RANKL to prevent its interaction with RANK, thereby inhibiting OCs formation and activity (Udagawa et al., 2021).

In healthy bones, the expression levels of RANKL and OPG are meticulously balanced to sustain optimal bone health. Following osteoclastic resorption, these cells release stored growth factors such as TGF-β, IGF-1, and BMPs. These factors serve to attract OPCs to the resorption sites and facilitate their differentiation into OBs, thereby promoting bone formation and completing the process known as “bone resorption-bone formation coupling.” In pathological conditions, an increase in RANKL or a decrease in OPG causes an elevated RANKL/OPG ratio, leading to excessive OCs activity that resorbs bone abnormally—especially on the inner surfaces of cancellous and cortical bones. This process results in the formation of Howship’s lacunae, which OBs are unable to adequately repair as a single-layer cover. Consequently, this leads to significant loss of the bone matrix, microstructural damage such as trabecular thinning and fractures, cortex thinning, increased porosity, a reduction in osteogenic scaffolding, and decreased mechanical stimulation (Kim et al., 2020; Martin and Sims, 2015; Robling and Bonewald, 2020; Chi et al., 2023).

Pathological bone resorption significantly disrupts the local microenvironment that sustains the “bone resorption-bone formation coupling.” This disruption leads to the accelerated degradation or inactivation of local growth factor reserves, thereby impeding the effective initiation and sustenance of osteogenesis. Moreover, inflammatory mediators originating from conditions such as inflammatory diseases or tumor-derived factors in cases of bone metastases can directly inhibit OBs differentiation and function or even induce apoptosis. Elevated levels of RANKL may also indirectly impair osteoblast lineage differentiation and activity. Collectively, these factors contribute to a rate of bone resorption that exceeds bone formation, resulting in a net loss of bone mass and potentially culminating in osteopenia or osteoporosis (Kim et al., 2020; Martin and Sims, 2015; Robling and Bonewald, 2020; Chi et al., 2023).

Mechanical stimulation is essential for regulating the RANKL/OPG balance, primarily through the activation of the Wnt/β-catenin pathway (Carrillo-López et al., 2021). Under standard mechanical loading conditions, RANKL levels diminish whilst OPG secretion elevates, thereby inhibiting OCs activity and bone loss, and consequently sustaining or enhancing bone mineral density (BMD) and facilitating bone formation. Conversely, mechanical unloading or atypical stimulation disturb this equilibrium, leading to an elevated RANKL/OPG ratio and augmented bone resorption (Tobeiha et al., 2020).

The RANKL/OPG ratio is reciprocally controlled by osteogenic pathways: Wnt/β-catenin signaling upregulates OPG to suppress bone resorption, while BMP-2 can transiently induce RANKL during early differentiation to facilitate coupled remodeling, illustrating how bone formation and resorption are molecularly interlocked. Table 1 summarizes the core signaling modules discussed in Section 4.

**TABLE 1:** This table summarizes the core signaling modules discussed in Section 4, highlighting their ligands, transduction mechanisms, temporal regulation, and mutual cross-talk during osteogenesis.

Signaling pathway	Core ligands	Receptors/Sensors	Key downstream effectors	Primary function in bone regeneration	Cross-talk with other pathways	Ref.
Wnt/β-catenin	Wnt3a, Wnt10betc.	Frizzled, LRP5/6	β-catenin, TCF/LEF, RUNX2, OSX	Low early, high late: early inhibition prevents premature osteogenesis; later robustly drives osteogenic differentiation and matrix synthesis	Synergistically activates RUNX2 with BMP/Smad; regulated by mechanical stimuli	(Tan et al., 2019; Huybrechts et al., 2020; Gao et al., 2023; Baron and Kneissel, 2013; Kang and Robling, 2015; Kitase et al., 2010; Tan et al., 2019; Huybrechts et al., 2020; Gao et al., 2023; Baron and Kneissel, 2013; Kang and Robling, 2015; Kitase et al., 2010)
BMP/Smad	BMP-2/4/7etc.	BMPR-I/II	Smad1/5/8, Smad4, RUNX2, OSX	Gradient-mediated MSC recruitment, osteogenic lineage commitment, matrix mineralization, and positive feedback amplification	Synergizes with Wnt/β-catenin; competitively antagonized by TGF-β/Smad2/3	(Wang et al., 2010; Zou et al., 2021; de Ceuninck van Capelle et al., 2020; Fang et al., 2016; Miyazawa and Miyazono, 2017; Kim et al., 2021; Kaur et al., 2021; Pang et al., 2022; Rawadi et al., 2003; Mbalaviele et al., 2005; Wu et al., 2024)
TGF-β/Smad2/3	TGF-β1/2/3	TGF-βR	Smad2/3, Cyclin D1, Sox9	Early: promotes OPC proliferation and migration; late: inhibits BMP-driven terminal osteogenic differentiation	Competes for Smad4, thereby inhibiting BMP/Smad1/5 signaling	(Wu et al., 2016; Wu et al., 2024)
MAPK (e.g., ERK, p38)	Mechanical stimuli, growth factors	Integrins, Piezo1/2	Ras→Raf→MEK→ERK1/2, p38	Converts mechanical signals into biochemical cues; promotes OB proliferation, differentiation, and matrix synthesis	Integrates mechanical–biochemical signals together with Wnt and BMP	(Jang et al., 2022; Qin et al., 2021; Zhang et al., 2023; Hu et al., 2024; Dou et al., 2019; Thouverey and Caverzasio, 2012)
RANKL/OPG	RANKL (pro-resorption) vs. OPG (protective)	RANK (on OC precursors)	NFATc1, NF-κB, MAPK	Maintains bone resorption–formation coupling; imbalance leads to osteoporosis	Downstream TGF-β/BMP release influences OBs; co-regulated with Wnt by mechanical stimuli	(Honma et al., 2021; Udagawa et al., 2021; Martin and Sims, 2015; Robling and Bonewald, 2020; Chi et al., 2023; Carrillo-López et al., 2021; Tobeiha et al., 2020)

* This table summarizes the core signaling modules discussed in Section 3, highlighting their ligands, transduction mechanisms, temporal regulation, and mutual cross-talk during osteogenesis.

## 5 Regulatory role of secretory proteins in osteogenesis

Whereas Sections 2, 3 profiled the major secreted protein families and their downstream signaling pathways in relative isolation, this section examines their integrated effects on defined cellular outcomes: osteoblast proliferation, differentiation, matrix mineralization, and osteoclast regulation.

### 5.1 Secreted proteins regulate osteoblast growth

Secreted proteins facilitate OBs proliferation by regulating the cell cycle, preventing apoptosis of OBs, and initiating metabolic reprogramming. For example, insulin-like growth factor 1 (IGF-1) stimulates the PI3K/AKT/mTOR pathway in a dose-dependent manner, thereby enhancing osteoblastic mRNA translation (Bakker et al., 2016). FGF2 activates the PI3K/Akt pathway by inducing cytoplasmic asparagine-tRNA synthetase (NARS), which promotes the proliferation of MC3T3-E1 cells and primary mouse cranial cells (Park et al., 2009). Additionally, vitamin D binds to its receptor in OBs, activating the PI3K/Akt pathway, which leads to the phosphorylation of downstream molecules, thereby reducing caspase activity, decreasing apoptosis, and enhancing OBs survival (Zhang and Zanello, 2008). Overall, secretory proteins are crucial in the regulation of OBs growth and survival, offering promising targets and strategies for bone tissue engineering, regeneration, and associated diseases.

### 5.2 Secreted proteins modulate Osteoblast differentiation

Secreted proteins facilitate osteogenic differentiation through the activation of transcription factors such as RUNX2 and OSX or by modulating epigenetic modifications. BMP-2/7 interacts with receptors, prompting the phosphorylation of Smad1/5/8, which then forms a complex with Smad4. This complex translocates into the nucleus, binds to the promoters of RUNX2 and OSX, and initiates the expression of osteogenic genes (e.g., *ALP*, *COL1A1*) (Wiesli et al., 2025; Wang et al., 2010). In a rat model of open fracture, the administration of local BMP-2 injections effectively promotes healing, reduces the duration of open wound exposure during surgical procedures, and decreases the risk of infection (Cheng et al., 2016). Platelet-derived growth factor BB (PDGF-BB) is involved in multiple stages of bone repair through activation of the PDGF-BB/PDGFR-β pathway. During the later stages of bone healing, the inhibition of PDGFR-β results in an increase in Smad protein levels, suppresses the Erk 1/2 pathway, and promotes the differentiation of BMSCs. This suppression additionally enhances Smad protein expression and reduces ERK 1/2 activity, thereby facilitating the differentiation of BMSCs into osteogenic cells (Wang et al., 2023). DKK1 has the capacity to restore functionality and augment the osteogenic potential of BMSCs in patients with impaired bone marrow environments by reducing Wnt3a levels and inhibiting the Wnt/β-catenin signaling pathway (Qi et al., 2021). Reduced levels of TGF-β1 enhance the phosphorylation of Smad2/3 through the TGF-β1/Smad2/3 pathway, thereby inhibiting excessive proliferation and abnormal differentiation in human osteogenic sarcoma cells, while concurrently promoting their osteogenic differentiation (Liu et al., 2017). Secretory proteins constitute a regulatory network that governs osteogenic differentiation through the modulation of various signaling pathways.

### 5.3 Secreted proteins regulate bone matrix mineralization

Bone matrix mineralization is fundamental to osteogenesis, characterized by the methodical deposition of hydroxyapatite crystals on collagen fibers. This process is meticulously regulated by various secretory proteins that supervise the initiation and progression of mineralization. Their roles include maintaining calcium and phosphorus concentrations, facilitating crystal nucleation, and directing the assembly of the matrix.

ALP is a crucial secreted enzyme indispensable for osteogenesis as it modulates bone matrix mineralization. It catalyzes the hydrolysis of PPi to produce Pi, which serves as the phosphate substrate necessary for hydroxyapatite crystal development. This activity diminishes PPi’s inhibitory influence on crystallization and elevates the local pH, thereby establishing a more conducive environment for calcium-phosphorus deposition and consequently fostering bone mineralization (Cannalire et al., 2023; Vimalraj, 2020). Research indicates that ALP deficiency leads to hypophosphatasia (HPP), a condition characterized by osteomalacia, impaired mineralization, premature tooth loss, and markedly reduced ALP levels in serum and bone tissue (Seefried et al., 2025). These features highlight ALP’s essential function in preserving bone health and appropriate mineralization.

OCN represents the most abundant non-collagenous protein within the bone matrix, playing a crucial role in the regulation of mineralization and the maintenance of mechanical strength. Upon γ-carboxylation, OCN selectively binds calcium ions through glutamate residues, directing hydroxyapatite deposition along collagen fibers. This mechanism enhances the microstructural integrity and mechanical resilience of the bone matrix, enabling it to withstand physiological loads (Komori, 2020). Nonetheless, the biological functions of OCN are regulated by a variety of post-translational modifications. For instance, glycation changes OCN’s three-dimensional conformation, which diminishes its capacity to bind hydroxyapatite. This reduction in binding ability adversely affects bone functions such as energy dissipation, thereby decreasing toughness and elevating the risk of fractures (Bailey et al., 2023).

BSP is a bone matrix protein that fulfills multiple biological functions. Its molecular structure comprises an RGD (Arg-Gly-Asp) sequence, which promotes the effective adhesion of OBs to the bone matrix—an essential factor for preserving bone tissue integrity and facilitating intercellular communication. Moreover, sulfation-modified tyrosine residues within BSP exhibit specific binding affinity to hydroxyapatite, thereby assisting in the nucleation of mineralized cores during the initial stages of mineralization (Xu et al., 2017). Research indicates that BSP impacts the processes of clone formation, differentiation, and mineralization activity in mouse cranial OBs *in vitro*. Notably, a BSP-deficient bone microenvironment markedly obstructs the proliferation of early OPCs and affects their cell fate (Bouet et al., 2015).

OPN functions as a vital negative regulator of osteogenesis, effectively mitigating the overgrowth of hydroxyapatite crystals through the binding of calcium ions facilitated by its abundant aspartate and glutamate residues. This mechanism exerts a substantial inhibitory influence on bone mineralization. Significantly, the phosphorylation status of OPN substantially modulates its activity: phosphorylation notably enhances OPN’s ability to impede hydroxyapatite crystal development (Bai et al., 2022). Furthermore, it has been demonstrated that phosphorylated OPN induces apoptosis in chondrocytes and enhances the production of pro-inflammatory mediators, thereby expediting the progression of OA (Gao et al., 2016).

PPi serves as a potent natural inhibitor of mineralization, playing a crucial role in sustaining the equilibrium between mineralized and non-mineralized regions of bone tissue. It accomplishes this by competing for hydroxyapatite nucleation sites and hindering crystal growth. In human physiology, PPi levels are meticulously regulated through a sophisticated process primarily involving ALP and the transmembrane pyrophosphate transporter protein (ANKH). ALP diminishes mineralization inhibition by hydrolyzing PPi into Pi, whereas ANKH oversees the transport of PPi across cell membranes, thereby maintaining the concentration gradient within and outside cells (Molin, 2024). Additionally, PPi plays an important role in cell signaling. Research indicates that PPi can significantly inhibit osteogenic differentiation and mineralization of periodontal ligament stem cells (PDLSCs) *in vitro* by activating the MAPK signaling pathway, which includes key kinases like ERK1/2, JNK, and p38 (Liang et al., 2021).

### 5.4 Secreted proteins modulate osteoclast activity

In the bone regeneration microenvironment, a high RANKL/OPG ratio—characterized by diminished OPG production and RANKL overexpression—serves as a principal factor driving abnormal OCs activation. This imbalance initiates a cascade of pathological events: excessively active OCs degrade the bone matrix, while locally secreted TGF-β/BMP-2 facilitates fibroblast infiltration and fibrous scarring, which further inhibits OBs activity in a destructive cycle. Consequently, reducing the RANKL/OPG ratio—via the administration of anti-RANKL antibodies or OPG analogs—is crucial for reversing pathological bone loss. This approach inhibits RANKL signaling, thereby preventing NFATc1 translocation and activation within the nucleus, and decreases mitochondrial-derived reactive oxygen species (ROS), effectively inhibiting osteoclastogenesis and bone resorption. Such strategies are considered central targets for the prevention and treatment of osteoporosis (Hong et al., 2021). Furthermore, targeted upregulation of OPG expression in OBs to reestablish the OPG/RANKL equilibrium markedly improves femur microarchitecture and bone mineral density in postmenopausal osteoporosis rat models, thereby providing an osteoprotective benefit (Ni et al., 2023).

Macrophage colony-stimulating factor (M-CSF) activates the PI3K/Akt and ERK signaling pathways through its binding to the c-Fms receptor (CSF-1R) on osteoclastic precursor cells. This engagement initiates a signaling cascade that promotes cellular proliferation and increases RANK expression, thereby enhancing the responsiveness of osteoclastic precursors to RANKL (Kim and Kim, 2016). Research indicates that reducing M-CSF levels within the bone microenvironment can substantially diminish OCs formation and activity, thereby aiding in the prevention of osteoporosis (Zhao et al., 2019).

Interferon-gamma (IFN-γ) inhibits OCs fusion and differentiation by activating the STAT1 pathway and reducing NFATc1 expression. Furthermore, IFN-γ promotes the degradation of TRAF6 and suppresses RANKL signaling (Cheng et al., 2012). Research indicates that mice deficient in the IFN-γ receptor demonstrate decreased bone mass, modified bone microarchitecture, and diminished levels of markers associated with bone formation and resorption. Conversely, the administration of IFN-γ notably enhances the number of osteogenic and osteoclastic cells, thereby improving bone strength in ovariectomized (OVX) mice. This underscores the potential therapeutic role of IFN-γ in the prevention and treatment of osteoporosis (Duque et al., 2011). The key secreted proteins and their regulatory roles in osteogenesis are summarized in Table 2.

**TABLE 2:** Summary of key secreted proteins and their functional roles in bone regeneration.

Secreted protein/Family	Primary cellular source(s)	Key receptors/Pathways	Core functions in bone regeneration	Primary stage(s) of action	Ref.
BMP-2/4/7	Inflammatory cells, periosteal cells, OBs	BMPR-I/II → Smad1/5/8	MSC recruitment, osteogenic lineage commitment, matrix mineralization	Throughout, especially early and mid-to-late stages	(Aryal et al., 2014; Xiao et al., 2007; James et al., 2016; Bharadwaz and Jayasuriya, 2021; Wu et al., 2016)
FGF-2	Injured local cells, MSCs	FGFR1-4, PI3K/Akt	Chemotaxis, MSC proliferation, angiogenesis	Early inflammation, mid-repair	(Charoenlarp et al., 2017; Zhou et al., 2023; Yang et al., 2018; Zhang et al., 2020; Takeda et al., 2024)
FGF-18	OBs, chondrocytes	FGFR3, MAPK/PI3K-Akt	Endochondral ossification, cartilage matrix formation	Mid-repair	(Murugaiyan et al., 2023; Cinque et al., 2015)
FGF-23	Osteocytes	FGFR/αKlotho	Inhibition of mineralization, phosphate homeostasis	Late remodeling	Moe and Moe (2017)
VEGF-A	ECs, OBs, MSCs	VEGFR1/2	Angiogenesis, vascular-osteogenic coupling, stem cell recruitment	Throughout, driving vascularized regeneration	(Hu and Olsen, 2016; Pérez-Gutiérrez and Ferrara, 2023; Kaigler et al., 2006; Kusumbe et al., 2014; Tang et al., 2021; Guo et al., 2023; Peng et al., 2020; Grunewald et al., 2006)
IL-1β	M1 macrophages, neutrophils	NF-κB	Pro-inflammatory, inhibits OB function, promotes osteoclastogenesis	Early inflammation	(Polzer et al., 2010; Tseng et al., 2022)
IL-4/IL-13	Th2 cells, M2 macrophages	STAT6	M2 polarization, OB proliferation/differentiation, inhibited osteoclastogenesis	Mid-repair	(Iwaszko et al., 2021; Allen, 2023)
IGF-1	OBs, liver	PI3K/AKT/mTOR	OB proliferation, inhibition of apoptosis	Throughout	Bakker et al. (2016)
PDGF-BB	Platelets, ECs	PDGFR-β	Progenitor cell recruitment; late-stage promotion of BMSC osteogenic differentiation	Throughout, especially late stage	Wang et al. (2023)
DKK1	OBs, osteocytes	LRP5/6 (Wnt inhibition)	Early inhibition of Wnt to prevent premature osteogenesis	Early inflammation	(Huybrechts et al., 2020; Gao et al., 2023; Baron and Kneissel, 2013; Qi et al., 2021)
OCN	OBs	Ca^2+^ binding	Regulates hydroxyapatite deposition, enhances mechanical properties	Mineralization	(Komori, 2020; Bailey et al., 2023)
BSP	OBs	Hydroxyapatite binding, RGD–integrin	Promotes OB adhesion, mineral nucleation	Mineralization	(Xu et al., 2017; Bouet et al., 2015)
OPN	OBs, osteocytes	Ca^2+^ binding	Inhibits excessive mineralization, regulates crystal growth	Mineralization	(Bai et al., 2022; Gao et al., 2016)
ALP	OBs, OPCs	Hydrolyzes PPi	Provides Pi for mineralization, promotes crystal formation	Mineralization	(Cannalire et al., 2023; Vimalraj, 2020; Seefried et al., 2025)
RANKL	OBs, osteocytes	RANK	Promotes OC differentiation and bone resorption	Throughout bone remodeling	(Honma et al., 2021; Martin and Sims, 2015)
OPG	OBs	Decoy receptor binding RANKL	Inhibits OC formation	Throughout bone remodeling	(Udagawa et al., 2021; Martin and Sims, 2015)
M-CSF	OBs, stromal cells	c-Fms (CSF-1R)	Promotes OC precursor proliferation and RANK expression	OCs process	(Kim and Kim, 2016; Zhao et al., 2019)
IFN-γ	Immune cells	STAT1	Inhibits OC fusion, downregulates NFATc1	OCs modulation	(Cheng et al., 2012; Duque et al., 2011)

* This table provides a panoramic overview of the major secreted proteins and their functional involvement across the sequential stages of bone regeneration, encompassing both regulatory and effector molecules that coordinate MSC, recruitment, osteogenic differentiation, matrix mineralization, and bone remodeling.

## 6 Clinical applications and potential of secretory proteins in bone formation

Before examining specific therapeutic strategies, it is instructive to consider how the secretory network becomes dysregulated in common bone disorders. In diabetic bone defects, chronic hyperglycemia and AGE accumulation suppress MSC responsiveness to BMP-2 and FGF-2, while persistent inflammation delays the M1-to-M2 macrophage transition required for repair (Bugger and Abel, 2014). In postmenopausal osteoporosis, estrogen loss elevates the RANKL/OPG ratio and upregulates the Wnt antagonist sclerostin, uncoupling bone resorption from formation (Sabry et al., 2021). In fracture nonunion, premature attenuation of the BMP gradient and insufficient VEGF-driven angiogenesis collectively prevent establishment of a functional osteogenic niche (Orth et al., 2022). These disorder-specific secretory defects provide the mechanistic rationale for the protein-based therapeutic strategies discussed below.

### 6.1 Bone tissue engineering strategies based on secreted proteins

The primary challenge in diminishing the regenerative capacity of bone with age or in conditions such as diabetes and inflammation is the disruption of the precise temporal and spatial regulation of secreted proteins. Within the realm of bone tissue engineering, a prevalent approach involves the incorporation of secreted proteins onto biomaterial scaffolds to facilitate osteogenesis and angiogenesis through synergistic effects. Present research emphasis is placed on two principal areas: the regulation of osteogenic differentiation and the elucidation of vascular-osteogenic coupling mechanisms. Significantly, BMP/Smad and Wnt/β-catenin signaling pathways are integral to the control of MSCs’ osteogenic differentiation. In the context of vascularized bone regeneration, VEGF and hypoxia-inducible factor 1-alpha (HIF-1α) serve as pivotal regulators. Empirical studies demonstrate that the co-application of VEGF and BMP-2 in a three-dimensional culture environment can augment VEGF’s capacity to upregulate BMPR expression on cellular surfaces, thereby increasing BMP-2 binding sites and markedly enhancing the dentinogenic differentiation of dental pulp stem cells (DPSCs) (Aksel et al., 2018). Furthermore, the activation of HIF-1α enhances endogenous VEGF levels, thereby facilitating the osteogenic differentiation of BMSCs and contributing to the preservation of alveolar bone density (Liu and Li, 2024).

Utilizing delivery systems such as nanoparticles, EVs, or hydrogels to regulate the sustained release of secreted proteins is crucial for enhancing their efficacy. Research indicates that PLGA nanoparticle systems are capable of delivering small doses of BMP-2 over an extended duration. PLGA microspheres encapsulating BMP-2 significantly augment ALP activity and calcium deposit formation in BMSCs, thereby demonstrating their ability to induce osteogenic differentiation (Minardi et al., 2020). Exosome-mediated BMP-2 markedly elevates the expression of proteins involved in tendon regeneration and cartilage formation through the Smad/RUNX2 pathway, thereby facilitating tendon-bone healing in rotator cuff injury models (Han et al., 2022). Furthermore, the carboxymethyl chitosan-based hydrogel system affords on-demand BMP-2 release, with its sol-gel transition meticulously synchronized with the evolving requirements of the bone repair process (Zhou et al., 2024).

In the field of bone tissue engineering, current research emphasizes multifactorial and synergistic strategies such as time-sequential release systems and the cross-regulation of signaling pathways. In a mouse ectopic bone formation model, a composite delivery system was utilized to sequentially release BMP-2 and VEGF: the initial release of BMP-2 promptly initiates osteogenesis, while subsequent VEGF release supports ongoing angiogenesis and bone maturation (Zhang et al., 2019). The synergistic regulation of FGF/ERK and BMP/Smad pathways effectively coordinates OBs proliferation and differentiation, thereby enhancing osteogenic development (Taketomi et al., 2018). Furthermore, innovative protein engineering and gene editing technologies are employed to design gene-activating proteins. For instance, the knockdown of the *NOG* gene, an antagonist of the BMP pathway in C2C12 cells, successfully increased BMP-2 signaling, thereby promoting OBs differentiation and mineralization (Çakmak et al., 2023).

In bone tissue engineering, secreted proteins are increasingly transitioning from research to clinical use. BMP-2 (Infuse®) is now approved for spinal fusion and tibial fracture treatment. Clinical studies show BMP-2 (Infuse®) achieves fusion rates comparable to traditional autologous iliac bone grafts, while also removing the need for bone removal surgery—shortening operative time and decreasing intraoperative bleeding. Despite promising results, limited clinical data mean its full efficacy requires further study (Fitzgerald et al., 2025). Additionally, recombinant human platelet-derived growth factor-BB (rhPDGF-BB, GEM 21S®) has been approved for periodontal bone defect repair. Clinical outcomes indicated significant reduction in probing depth at 6 months post-treatment; by 1 year, the depth stabilized between 4 mm and 6 mm, with neoplastic bone filling in the defect area reaching 85%–95% (Batra et al., 2023).

### 6.2 Therapeutic strategies for secretory protein-targeted osteoporosis

Osteoporosis treatment primarily involves using secreted proteins to balance bone formation and resorption. Key signaling pathways and targets include the RANKL/RANK/OPG, Wnt/β-catenin, and BMP/Smad pathways. To enhance systemic and local delivery, researchers have employed methods like nanocarriers, hydrogel slow-release systems, and exosome-mediated delivery. For instance, IGF-1 in PLA microspheres can provide sustained release for up to 180 days, targeting bone tissues and reducing articular cartilage breakdown in adult and aged mice (Liu et al., 2018). Similarly, hyaluronic acid hydrogels enable long-term BMP-2 delivery, significantly increasing implant fixation strength in osteoporosis models, promoting new bone growth around the implant, and offering improved bone support for patients (Siverino et al., 2024). MSCs-derived exosomes silenced the *SHN3* gene through siRNA delivery, while also promoting osteogenic differentiation, inhibiting OCs formation, and stimulating H-type angiogenesis—providing a comprehensive, multi-targeted approach to osteoporosis treatment (Cui et al., 2021). Gene editing and engineered proteins are important focus areas in this field. Using CRISPR/Cas9 to delete OCN in a rat model led to notable increases in cancellous bone volume, bone volume fraction, trabecular thickness, and bone mineral density. This reinforces the concept that OCN functions as a negative regulator of bone formation and suggests a new avenue for gene therapy in osteoporosis (Lambert et al., 2016).

Currently, the approved protein drugs for osteoporosis are Denosumab and Romosozumab. Denosumab, a monoclonal antibody, targets RANKL to inhibit osteoclastogenesis by blocking RANKL itself. It is prescribed for postmenopausal women and men with osteoporosis. After a year of treatment, patients showed a significant increase in BMD at the femoral neck and spine, along with a noticeable reduction in fracture risk (Kwon et al., 2024). Romosozumab is another monoclonal antibody that targets sclerostin, thereby activating the Wnt signaling pathway through neutralization of sclerostin. It is recommended for patients with severe osteoporosis who have a high fracture risk. After 12 months of therapy, these patients experienced a significant increase in spinal BMD, especially those with severe disease, low initial spinal BMD, and elevated levels of bone turnover markers TRACP-5b (OCs activity) and iP1NP (OBs activity). (Tominaga et al., 2021).

Looking ahead, the convergence of gene editing, synthetic biology, and artificial intelligence (AI) holds transformative potential for bone regenerative therapeutics. CRISPR/Cas9-mediated deletion of negative regulators suggests that the permanent reprogramming of bone cells’ secretory output is feasible. Moreover, AI-driven protein design may enable the engineering of synthetic growth factors with enhanced receptor selectivity and reduced adverse effects, while machine learning algorithms trained on multi-omics datasets could predict individualized combinations, doses, and temporal sequences of secretory factors for personalized bone repair.

### 6.3 Therapeutic approaches for fracture healing mediated by secretory proteins

Effective fracture repair depends on the precise timing and location of secreted proteins that promote bone healing. Key regulatory proteins involved are BMPs, VEGF, FGF-2, and PDGF. Utilizing advanced delivery systems and staged release techniques is vital for this process. Research shows that delivering BMP-2 and SDF-1α sequentially through nanocapsules significantly boosts new bone formation at the fracture site, resulting in higher bone volume and trabecular thickness compared to controls in a de-ovulated osteoporotic rat model, thereby improving fracture healing (Sun et al., 2023). Additionally, a time-sequential release system was able to deliver two pro-angiogenic growth factors, VEGF and PDGF, simultaneously with low-dose BMP-2. This combination promoted microvascular growth and vascular network formation, significantly speeding up the healing process in a composite bone-muscle injury model. This approach offers a new strategy for repairing complex tissue injuries (Subbiah et al., 2021). Gene therapy offers a key reason for enhancing fracture repair: delivering the *COLIA1* gene into OBs using adenoviral vectors boosted ALP activity and promoted calcium nodule formation. In a de-ovulated rat tibial fracture model, this approach successfully increased COL I expression in the bone tissue and sped up healing (Zeng et al., 2018).

RhPDGF-BB (Augment®) is approved for ankle fusion and encourages the migration of osteogenic precursor cells by activating the PI3K/Akt pathway. Clinical results indicated that the group treated with the rhPDGF-BB/β-TCP-COL composite had a significantly higher joint fusion rate than the autologous bone graft group, with a shorter average fusion time. Patients reported no graft site pain, confirming its safety and effectiveness (Daniels et al., 2015).

Future fracture repair strategies will likely move beyond single-factor supplementation toward the spatiotemporally controlled delivery of secretory factor combinations that mimic the natural healing cascade, increasingly leveraging smart biomaterials and personalized, data-driven protocols. A comprehensive overview of the key secretory proteins, delivery strategies, and clinical applications discussed in this section is provided in Table 3.

**TABLE 3:** From mechanism to clinic: delivery systems and applications of secretory proteins for bone repair.

Application area	Research focus	Key secreted protein/Target	Delivery system/Technology	Ref.
Bone Tissue Engineering	Synergistic Angiogenesis & Osteogenesis	VEGF, BMP-2	3D Co-culture	Aksel et al. (2018)
	Hypoxic Microenvironment Regulation	HIF-1α, VEGF	Endogenous Activation	Liu and Li (2024)
	Sustained Growth Factor Delivery	BMP-2	PLGA Nanoparticles	Minardi et al. (2020)
	Exosome-Mediated Repair	BMP-2	Exosomes	Han et al. (2022)
	Smart Responsive Release System	BMP-2	Carboxymethyl Chitosan Hydrogel	Zhou et al. (2024)
	Sequential Release Strategy	BMP-2, VEGF	Composite Delivery System	Zhang et al. (2019)
	Signaling Pathway Crosstalk	FGF, BMP	-	Taketomi et al. (2018)
	Gene Editing to Enhance Signaling	BMP-2	Gene Knockdown (Noggin)	Çakmak et al. (2023)
Osteoporosis Therapy	Long-Term Growth Factor Delivery	IGF-1	PLA Microspheres	Liu et al. (2018)
	Local Enhancement of Osseointegration	BMP-2	Hyaluronic Acid Hydrogel	Siverino et al. (2024)
	Exosome-Based Multi-Target Therapy	SHN3 (siRNA)	MSC-Derived Exosomes	Cui et al. (2021)
	Gene Editing Therapeutic Exploration	OCN	CRISPR/Cas9	Lambert et al. (2016)
Fracture Healing	Sequential Release for Enhanced Repair	BMP-2, SDF-1α	Nanocapsules	Sun et al. (2023)
	Synergistic Repair of Composite Injury	VEGF, PDGF, BMP-2	Time-Sequential Release System	Subbiah et al. (2021)
	Gene Therapy to Enhance Osteogenesis	COLIA1	Adenoviral Vector	Zeng et al. (2018)

* This table summarizes representative strategies and evidence for secreted protein-based bone repair. While the majority remain preclinical, they collectively illustrate the trajectory toward spatiotemporally controlled, multi-factor delivery systems that increasingly inform clinical translation.

## 7 Conclusion

Secretory proteins have emerged as pivotal mediators of osteogenic regulation, with growing recognition of their translational potential in clinical bone regeneration. These proteins orchestrate bone repair and remodeling by dynamically modulating osteoblast and osteoclast activity through precisely coordinated spatiotemporal gradients and integrated signaling networks. Despite substantial mechanistic advances, clinical translation remains hindered by persistent challenges, including the need for enhanced biosafety profiles, improved delivery fidelity and pharmacokinetic control, and robust interdisciplinary frameworks to bridge bench-to-bedside gaps.

To accelerate therapeutic innovation, future research should focus on five strategic priorities. (1) Spatiotemporally programmed release systems: development of hierarchical biomaterials that recapitulate the physiological sequence of inflammation, repair, and remodeling by delivering phase-specific secretory factor cocktails with precise temporal resolution. (2) Systematic combinatorial optimization of secretory factors: adoption of high-throughput screening platforms to identify synergistic multi-factor combinations and empirically validated optimal dose ratios, moving beyond reductionist single-factor paradigms. (3) Single-cell and spatially resolved transcriptomic mapping of the bone healing secretome: generation of high-resolution spatiotemporal atlases to delineate cell-type-specific secretion dynamics, microanatomical protein gradients, and previously uncharacterized regulatory nodes during bone regeneration. (4) Targeted gene-editing strategies for endogenous osteogenic potentiation: application of CRISPR/Cas9 and next-generation editing tools to selectively activate pro-osteogenic pathways or repress key inhibitors (e.g., *SOST*, *NOG*) in skeletal lineage cells, with emphasis on stringent *in vivo* targeting specificity, off-target mitigation, and delivery safety. (5) AI-integrated computational frameworks for personalized secretory regimens: fusion of multi-omics datasets (genomic, transcriptomic, proteomic) with imaging-derived features and interpretable machine learning models to predict patient-specific factor compositions, dosing schedules, and release kinetics, enabling data-driven precision bone therapeutics.

Collectively, the convergence of stimuli-responsive biomaterials, precision genome editing, and AI-augmented design promises to transform bone regeneration into a more predictable, individualized, and resource-efficient therapeutic modality. This integrative paradigm holds significant promise for addressing high-unmet-need indications—including large-segment bone defects, progressive osteoporosis, and disuse-induced bone loss in microgravity environments—thereby advancing both fundamental skeletal biology and clinical orthopedic practice.
